# Enhanced Evaporation Strength through Fast Water Permeation in Graphene-Oxide Deposition

**DOI:** 10.1038/srep11896

**Published:** 2015-06-23

**Authors:** Wei Li Tong, Wee-Jun Ong, Siang-Piao Chai, Ming K. Tan, Yew Mun Hung

**Affiliations:** 1 Mechanical Engineering Discipline, School of Engineering, Monash University, 47500 Bandar Sunway, Malaysia; 2 Multidisciplinary Platform of Advanced Engineering, Chemical Engineering Discipline, School of Engineering, Monash University, 47500 Bandar Sunway, Malaysia

## Abstract

The unique characteristic of fast water permeation in laminated graphene oxide (GO) sheets has facilitated the development of ultrathin and ultrafast nanofiltration membranes. Here we report the application of fast water permeation property of immersed GO deposition for enhancing the performance of a GO/water nanofluid charged two-phase closed thermosyphon (TPCT). By benchmarking its performance against a silver oxide/water nanofluid charged TPCT, the enhancement of evaporation strength is found to be essentially attributed to the fast water permeation property of GO deposition instead of the enhanced surface wettability of the deposited layer. The expansion of interlayer distance between the graphitic planes of GO deposited layer enables intercalation of bilayer water for fast water permeation. The capillary force attributed to the frictionless interaction between the atomically smooth, hydrophobic carbon structures and the well-ordered hydrogen bonds of water molecules is sufficiently strong to overcome the gravitational force. As a result, a thin water film is formed on the GO deposited layers, inducing filmwise evaporation which is more effective than its interfacial counterpart, appreciably enhanced the overall performance of TPCT. This study paves the way for a promising start of employing the fast water permeation property of GO in thermal applications.

Since the early discovery of graphene in 2004^1^, it has attracted substantial interest due to its unique electrical, physical and thermal properties. The honeycomb lattice arrangement of the carbon atoms with a single atomic layer thick structure enables the superior thermal conducting characteristics of graphene. The thermal conductivity of a single layer graphene is reported ranging from 4840 W/m·K to 5300 W/m·K, outrunning that of carbon nanotube^2^. Despite an extensive amount of research work devoted to graphene, only a handful of studies related to its thermal applications have been reported^3^,^4^,^5^. The poor thermal interaction between graphene layer and adjacent substrate^6^ is a hindrance to the development of thermal applications but the biggest deterrent has undoubtedly been the complex synthesis method in producing large area of graphene sheet^4^,^5^,^7^. While graphene is one single layer of graphite, graphene oxide (GO), a functionalized graphene derivative which contains flakes of monolayer and few-layered graphene, can be prepared with a gentle sonication of graphite oxide in various solvents for facile and mass production^8^,^9^. In light of the presence of oxygen functionalities, GO has excellent solubility and stable dispersion in water and other organic solvents while retaining some of the superior properties of graphene^10^,^11^.

Recently, fast water vapor transport across laminated GO membrane while blocking even the smallest gas atom of helium has been discovered by Geim and co-workers^12^. Anomalously high permeation of water through GO laminates having vacant spaces formed between non-oxidized regions of graphene sheets was observed. Two factors contribute to this unusual water permeation – the capillary driven force and the remarkable boundary slip of ultralow-friction passage. The oxidized regions act as spacers to form a network of nanocapillaries that vigorously interact with the intercalating water while the two-dimensional graphene nanocapillaries allow low-friction flow of monolayer water. Water flowing through the empty region between the pristine graphene sheets experiences an ultra-fast, low-friction flow similar to those reported in the water permeation of carbon nanotubes^13^. The fast water transport confined in the nanocapillary is ascribed to the frictionless interaction between the atomically smooth, hydrophobic carbon wall and the well-ordered hydrogen bonds of water molecules^12^,^14^,^15^. For GO membranes immersed in liquid water, the mechanism of fast water permeation is not limited to non-oxidized regions^16^, based on the fact that no interconnecting network forms in these regions for water permeation^17^. Water intercalation through an immersed GO membrane engenders expansion of the interlayer distance between the graphitic planes^16^,^18^,^19^. As the interlayer distance expanded over 9–10 Å, the first water monolayer was found to be attached firmly to the oxidized region while the second water monolayer manifests its rapid movement in a translational motion^20^,^21^. The behavior of water intercalation of GO membranes was observed to be similar to that of GO powders^16^,^21^. It was also suggested that fast water permeation could be attributed to the permeation across defective holes laid across the GO sheets^16^. By virtue of vigorous oxidation of precursor graphite and sonication treatment, GO sheets were introduced with high percentage of defective sites, allowing the permeation of water molecules across the graphitic plane. The mechanism of fast water permeation was also studied *via* theoretical modeling^12^,^22^,^23^. It is yet conclusive to fully comprehend the underlying physical phenomenon of the anomalous fast water permeation of GO.

In view of the excellent properties measured in experiments, GO is a promising candidate for replacing metallic oxide nanoparticles in the preparation of nanofluid – a fluid containing nanoparticles suspensions^24^,^25^. Characterizations of graphene based nanofluid show extraordinary thermal conductivity enhancement with a small dosage of graphene^26^,^27^. The choice of working fluid has been a key area of interest in enhancing the performance of cooling devices. The exponential growth in heat generation of electronics devices in light of the trend of multi-functionalization and miniaturization necessitates development of highly efficient cooling devices. Two-phase closed thermosyphon (TPCT) has been used extensively for thermal management due to its excellent heat transport capability and reliability^28^,^29^. A TPCT is essentially an evacuated tube charged with volatile working fluid, divided into three sections: evaporator, adiabatic section, and condenser. Working on the principle of phase-change heat transfer, the working fluid in the TPCT evaporates and absorbs latent heat from the heated evaporator section. The resultant vapor travels upwards through thermal buoyancy and condensation occurs at the sub-cooled condenser wall by releasing the latent heat to the surroundings. The condensate is then circulated back to the evaporator section *via* gravitational force and the cycle is perpetuated. The thermal efficiency of a TPCT is thus governed by the phase-change heat transfer processes namely evaporation and condensation, as well as circulation of condensate. As the suspension of nanoparticles in a base fluid is deemed to enhance the thermophysical properties, nanofluid was utilized as working fluid to improve the performance of TPCT^30^,^31^,^32^. However, the increase in thermal conductivity of nanofluid is not the sole factor in contributing heat transfer enhancement for phase-change processes, other predominant factors are also prevalent. Prior studies showed that nanoparticles agglomerate and segregate from the base fluid, forming thin porous layers on the inner surface of the evaporator^31^,^33^,^34^. In general, nanoparticles deposition on the heated surface is found to have significantly enhanced the surface wettability and nucleate boiling mechanism, thus increasing the evaporating heat transfer coefficient^35^,^36^,^37^,^38^. The performance augmentation of nanofluid-filled TPCT is elementally attributed to the surface modification of nanoparticles deposition instead of the increase in thermal conductivity^31^.

In this work, we introduce GO/water nanofluid as a working fluid to enhance the performance of a mini TPCT. As TPCT is a phase-change heat transfer device, its performance is dominated by evaporation process which occurs from a heated surface where a liquid vaporizes into a gaseous phase. The latent heat of vaporization is absorbed through the evaporator wall surface and the evaporation strength varies depending on the condition of the surface^39^,^40^. For the sake of maintaining high evaporation rate and achieving a high heat transfer rate, high surface temperature, large surface area, and low intermolecular strength of molecules are favorable conditions. Due to the distinguished physical properties of GO, the mechanism of evaporative heat transfer enhancement from GO deposition is hypothesized to be distinct from that of conventional metallic or non-metallic oxide nanoparticles. The fast water permeation property of GO inspires its application in enhancing the evaporation via surface functionalization. The GO deposition on the evaporator surface provides nanocapillaries (interconnecting networks at nanoscale) that draw the liquid water off the liquid pool to the sides of wall, giving rise to a larger surface area for evaporation and facilitating a higher evaporation rate. Notably, at nanoscale, capillary force is more intense and dominates over gravitational force, allowing liquid to flow in an antigravity direction. By comparing the performance of GO nanofluids to that of metallic oxide nanofluids, we scrutinize the mechanism of heat transfer enhancement in TPCTs. In addition, based on the structural studies of GO membrane in water of prior investigations^12^,^16^,^19^, we are able to comprehend the heat transfer characteristics induced by different thicknesses of GO deposition. We illustrate the experimental setup in the following section, characterize the nanofluids by examining the effective thermal conductivity and viscosity, and demonstrate the effects of the anomalous characteristics of GO deposition in TPCTs.

## Methods

### Experimental Setup and Data Reduction

**Figure 1:**
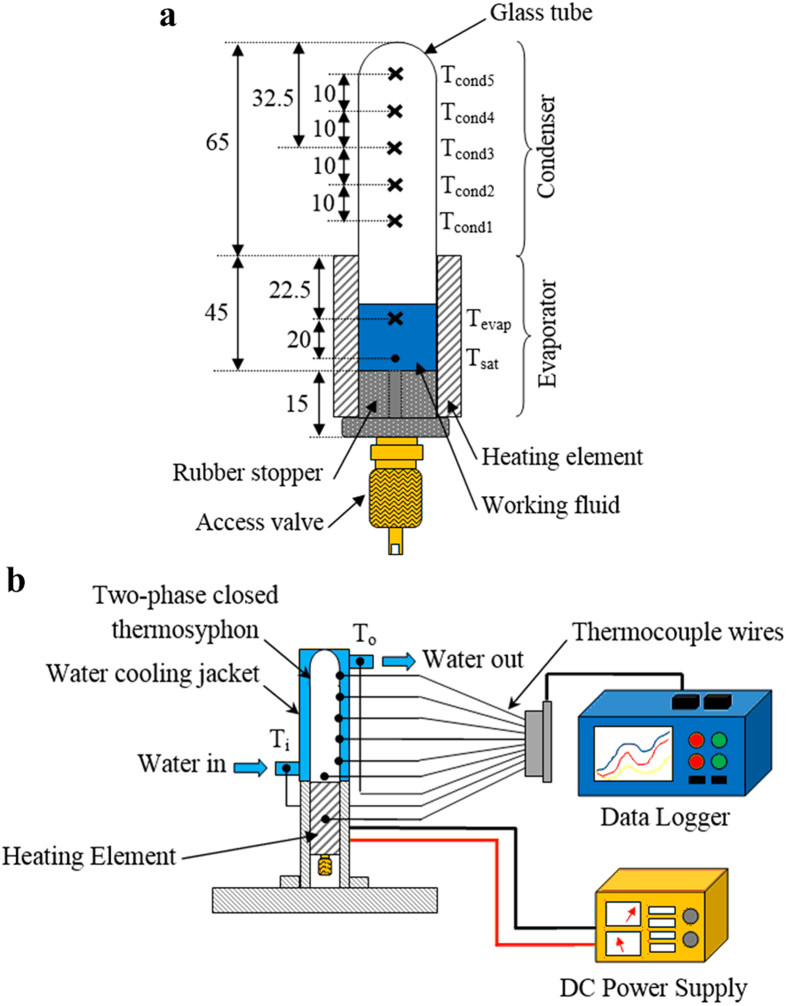
(**a**) Schematic diagram of a TPCT with temperature measurement points. (**b**) The experimental setup for the evaluation of performance of nanofluid charged TPCT.

A schematic diagram of the experimental setup is illustrated in Fig. 1. Briefly, the apparatus includes a TPCT, electrical heater element, water cooling jacket, data logger and direct-current (DC) power supply. The TPCT was fabricated using standard laboratory glass tube with an inner diameter of 13.5 mm and a length of 110 mm. The glass tube was sealed using a rubber stopper, which was embedded with an access valve. To ensure the glass tube was airtight, high strength epoxy was applied at all connections^32^. A 1 ml of working fluid, equivalent to 16.7% of fill ratio, was charged into the TPCT through the access valve. Two different types of working fluids were prepared: silver oxide (SO) nanofluids (solutions with weight ratios of 0.01% and 0.5%), and GO nanofluids (solutions with weight ratios of 0.01%, 0.025%, 0.05%, 0.075% and 0.1%). SO nanofluids refer to aqueous solutions with suspension of SO nanoparticles of diameter 30 nm (Sigma Aldrich), whereas GO nanofluids refer to aqueous solutions with suspension of graphite oxide. The graphite oxide was synthesized using the high purity graphite powder of size 45 μm (Sigma Aldrich); the protocol for synthesizing the graphite oxide powder is described in the next section, followed by the preparation of graphite oxide nanofluids. Once the charging was completed, the absolute pressure in the glass tube was reduced to 0.2 Pa using a vacuum pump. The evaporator section of the TPCT was in direct contact with a uniform electrical heating element, whereas the condenser section was cooled *via* a water cooling jacket, as shown in Fig. 1(b). The electric power input to the electrical heating element was controlled by adjusting the switch on the DC power supply. To minimize the heat loss from the electrical heating element to the surrounding, several layers of insulating materials were wrapped around the element. For performance analysis of the TPCT, the axial temperature distribution of the TPCT was measured using six type-T thermocouple wires, which were all connected to a data acquisition system. The liquid saturation temperature, *T*_sat_, was also measured by inserting a type-T thermocouple wire into the bottom section of the evaporator. In each test, the temperatures were recorded for a duration of 60 minutes and at a sampling rate of 2 readings per second.

Using the measured temperatures, the overall thermal performance can be characterized using the effective thermal resistance, given by


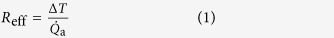


where 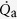 is the heat transport rate, Δ*T* = *T*_evap_ − *T*_cond_ is the axial temperature difference between the evaporator and the condenser, with *T*_evap_ the temperature at the evaporator and *T*_cond_ the temperature at the condenser. We note here that *T*_cond_ = (*T*_cond1_ + *T*_cond2_ + *T*_cond3_ + *T*_cond4_ + *T*_cond5_)/5, is the average temperature of the condenser and 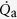 is calculated based on the principle of energy conservation, i.e., the net heat transported across the TPCT is equivalent to the heat dissipated from the condenser section, 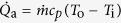, with the assumption of a well-insulated water cooling jacket. Two thermocouples are employed to measure the inlet and outlet water temperatures, T_i_ and T_o_. Here, 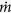 is the water mass flow rate and *c*_*p*_ is the specific heat capacity.

The average evaporator heat transfer coefficient, 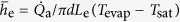, is used to quantify the strength of evaporation, where *T*_sat_ is the saturation temperature of the working fluid in the evaporator, *d* and *L*_e_ are the inner diameter and length of the evaporator, respectively. During the experiments, under a constant heat input, the evaporator heat transfer coefficient augmentation ratio is given by


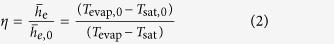


where 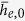 is the average heat transfer coefficient of deionized (DI) water charged TPCT. Here, *η* represents the relative comparison of the heat transfer coefficients by using the value of DI water charged TPCT as the basis for comparison. Enhancement in heat transfer at the evaporator can be noticed with *η* exceeding the value of one and vice versa.

### Synthesis of Graphite Oxide

Graphite oxide powder was synthesized *via* the modified Hummers’ method with the following procedures^41^,^42^. 3 g of graphite powder (Sigma Aldrich, <45 μm, > 99.99%) was added into an 80 °C mixture containing 12 ml of concentrate H_2_SO_4_ (Chemolab supplies, 95–97%), 2.5 g of P_2_O_5_ (Sigma Aldrich, ≥98%) and 2.5 g of K_2_S_2_O_8_ (Sigma Aldrich, ≥99%). The mixture was then stirred for 4.5 hours and cooled to room temperature before diluted with 500 ml of DI water. The mixture was continuously washed with DI water until the pH of the filtrate became neutral. The product was dried at 70 °C overnight. The pre-oxidized graphite was re-dispersed into 120 ml of cold concentrated H_2_SO_4_ together with 15 g of KMnO_4_ (Sigma Aldrich, ≥99%). The mixture was then stirred for 2 hours with the temperature kept below 20 °C. Next, the mixture was diluted with 250 ml of DI water in an ice bath to keep the temperature below 50 °C. After 2 hours of stirring, the solution was diluted again with 700 ml of DI water. 20 ml of H_2_O_2_ (R&M Chemicals, 30%) was further added into the final mixture which was then washed with 1 l of HCL (Merck, 37% diluted to 10%) followed by DI water for several times to completely remove the acid content. After filtration, the graphite oxide was air-dried at a temperature of 60 °C for 24 hours. The graphite oxide was then grounded into fine powder form. The X-ray diffraction (XRD) spectrum of GO powder indicates a peak at 9.1° corresponding to an interlayer distance of 9.72 Å, which is consistent with those of prior studies^16^,^19^.

### Preparation of GO and SO nanofluids

In order to obtain the final product of GO nanofluid, graphite oxide powder was dispersed in DI water and underwent ultrasonication treatment. Through sonication process, graphite oxide suspended in the aqueous solution exfoliates to yield a large distribution of nanometer-sized GO sheets. GO nanofluids were prepared with five concentrations measured by the weight of graphite oxide powder added to the dispersion (Supplementary Fig. S1). The weight percentages were maintained at 0.01 wt%, 0.025 wt%, 0.05 wt%, 0.075 wt% and 0.1 wt%. The mixtures were ultrasonicated (20 kHz, 700 W) with an ultrasonic liquid processor (Q700 Sonicator^®^, Qsonica, LLC.) for 5 hours with fluid temperature maintained below 80 °C to prevent evaporation. To quantify the superiority of the GO nanofluid, SO nanofluid was prepared in two concentrations of 0.01 wt% and 0.5 wt%. Similar ultrasonication treatment was used for the preparation of SO nanofluids to ensure the homogeneity.

### Characterization of GO and SO nanofluids

The heat transfer capability of a TPCT is governed by thermophysical properties such as the thermal conductivity and the viscosity of working fluid. The thermal conductivity of the nanofluid was measured using thermal property analyzer (KD2Pro, Decagon Devices, Inc., Canada) with an uncertainty of ±5%. The device measured the thermal conductivity using transient hot wire method with a probe sensor of 60 mm in length and 1.3 mm in diameter. Thermal bath was used to prevent temperature fluctuation and all samples were measured at the temperatures ranging from 25 °C to 45 °C. Prior to the taking of measurements, the KD2 Pro device was calibrated with glycerol. Ten measurements were taken for each sample at the targeted temperature to ensure the accuracy and reliability. The measured thermal conductivities of the GO and SO nanofluids were compared with thermal conductivities of the base fluid (DI water) measured under the same conditions.

**Figure 2:**
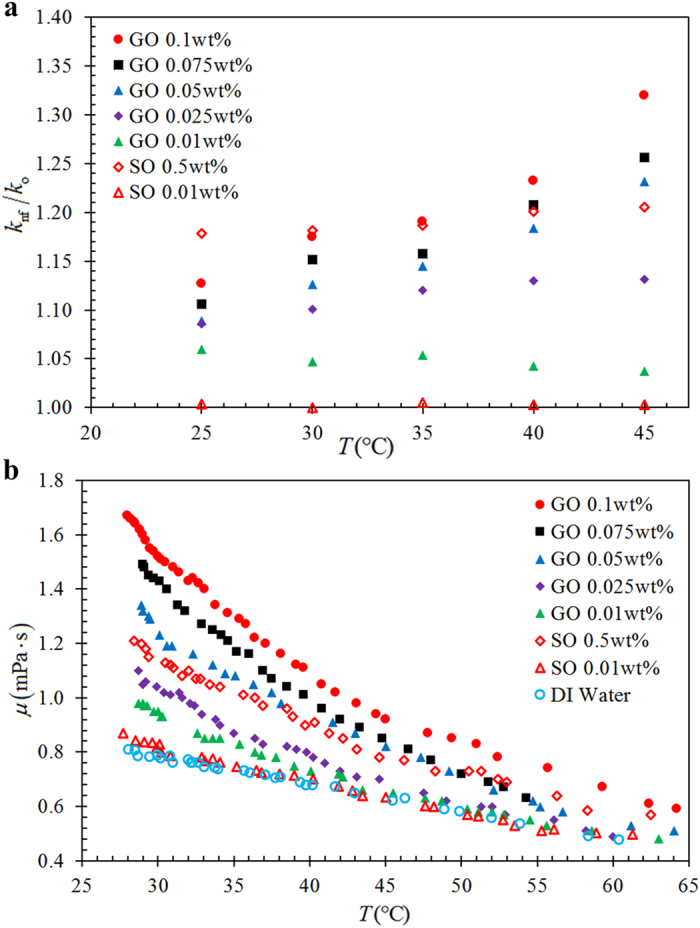
(**a**) Effective thermal conductivity enhancement ratio, and (**b**) viscosity, of GO and SO nanofluids as a function of temperature for different nanofluid concentration.

Figure 2(a) shows the thermal conductivity enhancement ratios of GO and SO nanofluids as a function of nanofluid concentration and temperature. The thermal conductivity enhancement ratio is defined as *k*_nf_/*k*_o_ where *k*_o_ is the thermal conductivity of the based fluid and *k*_nf_ is the thermal conductivity of the nanofluid. The SO nanofluid with 0.01 wt% has nearly no enhancement for the range of temperature from 25 °C to 45 °C. However, the GO nanofluids achieve an overall enhancement in thermal conductivity. Different trends in the change of enhancement ratio at different temperatures are observed for SO and GO nanofluids. At a high GO content of 0.1 wt%, the enhancement ratio increases exponentially. However, the enhancement ratio of 0.5 wt% SO nanofluid remains almost constant at different temperatures. The constant enhancement ratio with the increase of temperature implies that the base fluid has more dominant effect on the increase in thermal conductivity rather than the thermal transport behavior associated with the suspended nanoparticles. The thermal transport mechanisms such as micro-convection due to Brownian motion, ballistic phonon transport and clustering effect of nanoparticles are among those commonly affecting the increase in thermal conductivity of nanofluids^43^. Nevertheless, the factors affecting the thermal conductivity of GO nanofluids are distinguishable. For GO nanofluids, strong temperature dependence of thermal conductivity enhancement ratio is observed in the concentrations of 0.05 wt%, 0.075 wt% and 0.1 wt%. This can be attributed to the high thermal conductivity nature and the high surface area to volume ratio of GO sheets. As GO sheets have significantly larger contact area with the fluid molecules, the contact resistance at the graphene-fluid interface is substantially reduced. In light of high thermal conductivity nature of GO sheets, the thermal energy can be effectively transported across the solid-fluid interface, creating an excellent heat conduction path. Due to the high thermal conductivity and the 2D structure of GO sheets, a substantial thermal conductivity enhancement is attainable even at a low concentration of GO. On the other hand, the viscosity increases with concentration of nanoparticles. Figure 2(b) depicts the viscosities of the GO and SO nanofluids at different concentrations. The viscosity decreases with increasing temperature. At higher temperatures (above 60 °C), the viscosities of nanofluids rapidly decrease and approach the viscosity of base fluid (DI water). As the TPCT operates at temperatures higher than 60 °C, the effect of increase in viscosity on the thermal performance of TPCT can be deemed to be marginal.

### Surface Morphology of Nanoparticles Deposition

During the two-phase heat transfer process, a thin layer of nanoparticles was observed depositing on the heated evaporator surface. A variation in color or color intensity of the deposition was clearly noted for different weight percentages and different nanofluids. Samples of the deposition were prepared by gently breaking the glass structure of TPCT to obtain the wall of evaporator section where the deposition took place. To assure the consistency of the results, the samples were obtained at a location 5 mm from the bottom of the evaporator. Observation of surface morphology of deposited layers was carried out using a SU-8010 field emission scanning electron microscope (FESEM, Hitachi Ltd., Japan). Static contact angle *θ*_s_ between DI water and the deposition was measured using a standard goniometer (Model 590, Ramé-Hart Instrument Co.) under atmospheric pressure and a room temperature of 26 °C.

## Results and Discussion

### Performance comparison of GO and SO nanofluids

**Figure 3:**
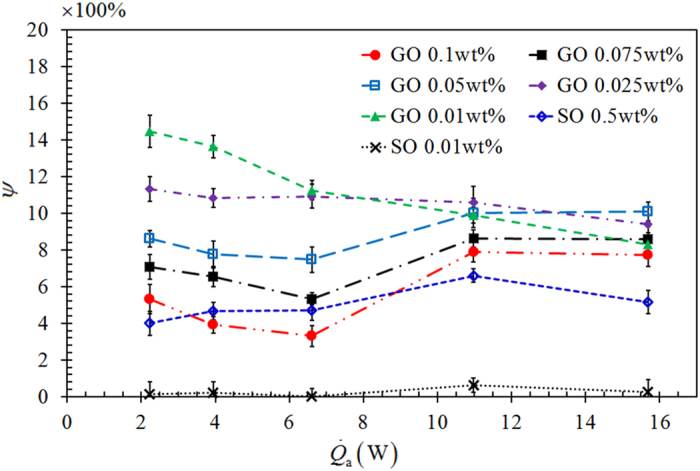
Variations of Δ*T* reduction ratio, *ψ* = (Δ*T*_nf_ − Δ*T*_o_)/ΔT_nf_, of GO and SO nanofluids charged TPCTs as a function of 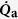, with the nanofluid concentration being a parameter.

The temperature difference, Δ*T* = *T*_evap_ − *T*_cond_, manifests itself as a convenient indicator in quantifying the heat transport rate along the axial direction. In accordance with the Fourier’s law of heat conduction, under the same heat transfer rate, smaller Δ*T* indicates higher heat transport capability of the specimen. A low Δ*T* infers a low thermal resistance across the evaporator and condenser sections. We observe that Δ*T* of a GO-nanofluid charged TPCT is lower than that of a DI water charged TPCT. Figure 3 shows the variations of Δ*T* reduction ratio, *ψ* = (Δ*T*_nf_ − Δ*T*_o_)/Δ*T*_nf_, _o_f GO and SO nanofluids charged TPCTs as a function of 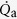, with the nanofluid concentration being a parameter. Here, Δ*T*_o_ is the temperature difference of the base fluid (DI water) charged TPCT, Δ*T*_nf_ is the temperature difference of the nanofluid charged TPCT, and *ψ* is regarded as a comparison of the change in Δ*T* of nanofluid charged TPCT with Δ*T* of a base fluid charged TPCT, indicating an enhancement in the performance of a nanofluid charged TPCT. The GO nanofluids charged TPCTs have higher Δ*T* reduction ratios than that of SO nanofluids charged TPCTs. At a very high concentration of SO (0.5 wt%), the performance of TPCT is comparable with that of 0.1 wt% GO nanofluid charged TPCT. This shows that the GO nanofluid TPCTs outperform the SO nanofluid ones. For GO nanofluid TPCTs charged with higher concentrations (0.05 wt%, 0.075 wt% and 0.1 wt%), we observe that *ψ* decreases with increasing 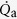 at low 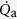 and starts to increase when 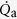 exceeds 6.5 W. This indicates that the GO nanofluid TPCTs perform better at a higher heat input, which will be discussed later.

**Figure 4:**
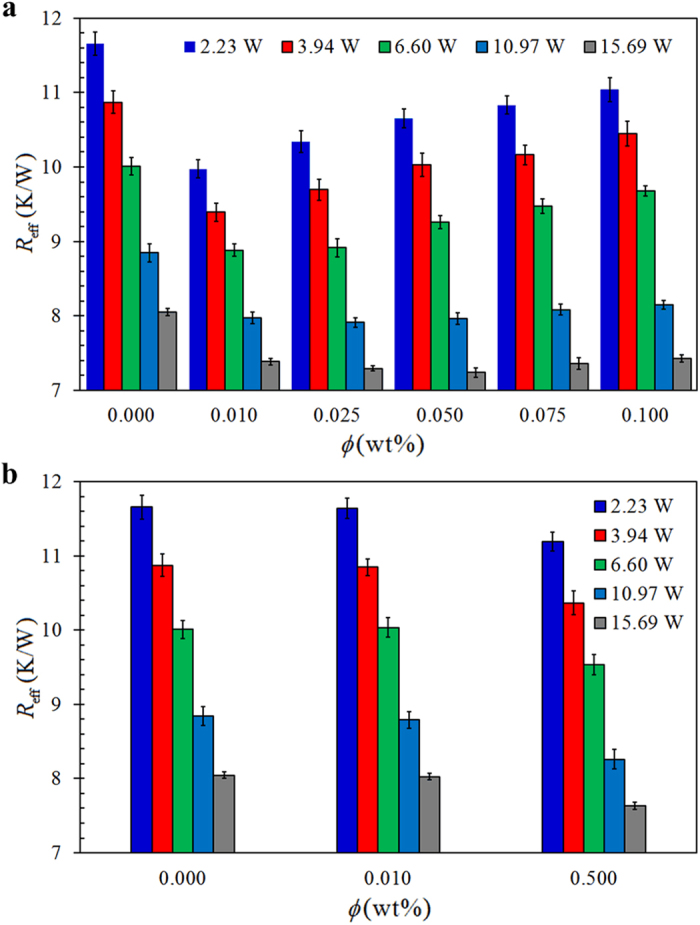
Effective thermal resistance, *R*_eff_, as a function of nanoparticles weight ratio, *ϕ*, of (**a**) GO nanofluids, and (**b**) SO nanofluids, charged TPCTs at different 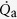.

To evaluate the overall performance of TPCT, the effective thermal resistance, *R*_eff_, which is a function of Δ*T* is analyzed. Figure 4a,b depict the variations of *R*_eff_ with 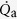, for different concentrations of GO and SO nanofluids charged TPCTs, respectively. The effective thermal resistance of the DI water (*ϕ* = 0) charged TPCT is used as a benchmark to illustrate the enhancement in thermal performance of nanofluid charged TPCTs. A lower *R*_eff_ indicates higher performance. The decrease in *R*_eff_ is essentially attributed to the enhancement of either evaporation strength at the evaporator or circulation effectiveness of condensed liquid back to the evaporator or of both. Basically the effective thermal resistance decreases with increasing 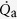. We observe that *R*_eff_ of GO nanofluid TPCTs is overall lower than that of DI water charged TPCT while *R*_eff_ of SO nanofluid TPCTs is only marginally lower than that of DI water charged TPCT even at high concentration of SO. Hence, comparatively the GO nanofluid TPCTs outperform the SO nanofluid TPCTs. Remarkably, for the case of GO nanofluids, *R*_eff_ increases with GO concentration *ϕ* at low 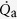 (2.23 W, 3.94 W and 6.6 W) and becomes independent of *ϕ* at high 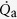 (10.97 W and 15.69 W). Referring to Fig. 2(a) which shows that the effective thermal conductivity of nanofluid increases with GO concentration, we expect that *R*_eff_ of TPCT should decrease with increasing GO concentration. However, the finding in Fig. 4(a) is contrary to what was anticipated based on the characterization of effective thermal conductivity of GO nanofluid as discussed in Fig. 2(a). In this regard, the reduction of *R*_eff_ is not entirely attributed to the increase in effective thermal conductivity of GO nanofluid. Other factors might have contributed to this unusual result. As mentioned, nanoparticles agglomerate in the base fluid and thin porous layers are formed on the inner surface of the evaporator. The nanoparticles deposition manifests significant enhancement in the surface wettability and nucleate boiling mechanism^31^,^33^,^34^. To this end, in what follows, we investigate the underlying physical significance of GO deposition and its anomalous characteristics in affecting the thermal performance of TCPT.

### Fast Water Permeation Property of GO Deposition

**Figure 5:**
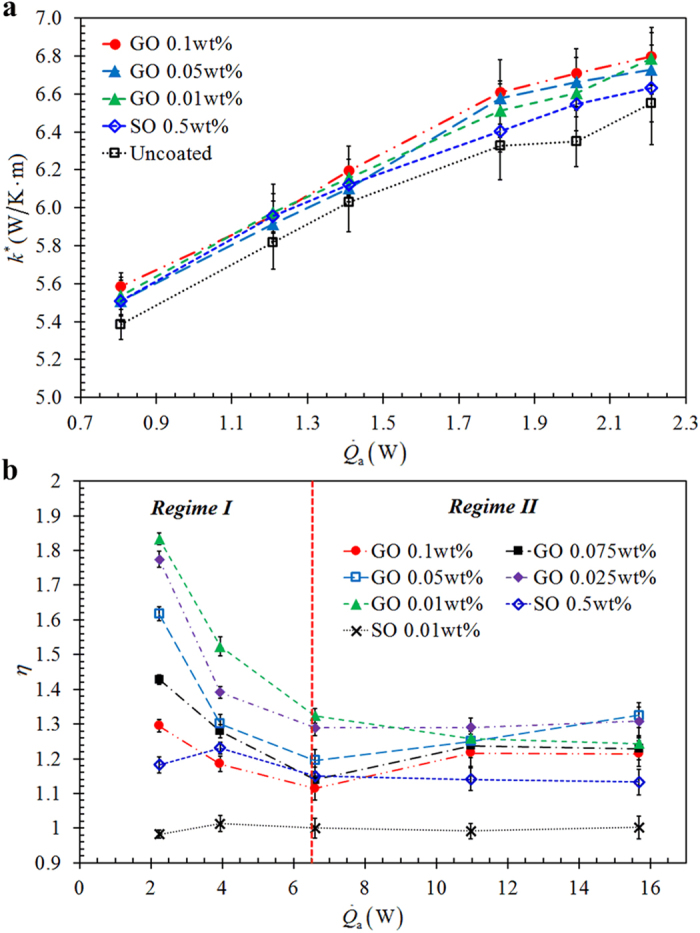
(**a**) Thermal conductance of uncharged TPCTs coated with thin GO and SO nanoparticles depositions as a function of 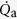 during the heat conduction experiments. (**b**) The evaporator heat transfer coefficient augmentation ratio, *η*, as a function of 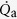 with nanoparticles weight ratio as a parameter. Two distinct regimes – 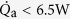 and 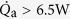 can be clearly identified.

To exclusively elucidate the role of thermal conductivity of nanoparticles deposition on the evaporator wall surface, we first examine the effective thermal conductance of uncharged TPCTs that had been coated with GO and SO during the experiments. The working fluids were evacuated from the TPCTs to exclude the two-phase heat transfer process. Simple heat conduction experiments were conducted. The primary objective is to examine the heat conduction contribution of the nanoparticle deposited layer. Except for the specimens, the experimental setup is identical to that in Fig. 1(b). The thermal conductance which can be considered as the effective thermal conductivity of specimen is calculated as 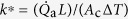, where *L* is the distance between the two measured temperatures, *A*_c_ is the cross section area of the evacuated glass tube and Δ*T* = *T*_evap_ − *T*_cond_ is the temperature difference. Figure 5(a) depicts the results of the heat conduction experiments. Although the thermal conductance of GO deposition is slightly higher than that of SO deposition, they are only marginally higher (with a maximum of 6%) than that of the uncoated surface. This is not surprising as GO intrinsically has significantly lower thermal conductivity as compared to the pristine graphene with high in-plane thermal conductivity nature^7^,^43^. Due to the introduction of oxygenated functional groups and defects in GO during vigorous oxidation process, in-plane heat transfer through lattice vibrations is impeded^7^,^44^. High in-plane thermal conductivity of graphene is attributed to the covalent sp^2^ bonding between the carbon atoms and the heat flow is anisotropic^7^. On the other hand, the layered structure of GO is governed by the cross-plane van der Waals force and the repulsive electrostatic force which is induced by the negatively charged functional groups^6^,^45^. As a result, the cross-plane heat transfer is ineffective as compared to the in-plane heat transfer. In fact, based on non-equilibrium molecular dynamics simulations, the thermal conductivity of GO with an oxygen coverage of 20% was estimated to be 8.8 W/m·K which is three orders of magnitude lower than the thermal conductivity of pristine graphene^43^. In addition, at the interface between the glass substrate and the adjacent GO sheets, poor van der Waals coupling limits the heat transfer^6^ and a high thermal resistance (hence a low thermal conduction) is induced between the GO sheets and the glass substrate. This shows the insignificant contribution of thermal conductivity of the GO deposition, which is essentially associated with heat conduction, to the thermal performance enhancement of TPCT. Hence we postulate that the performance enhancement and the effects of GO deposition are entailed by the two-phase heat transfer process.

As mentioned earlier, the two-phase heat transfer in a TPCT is governed by the evaporation process and the circulation of condensate. As the nanoparticles deposition takes place at the evaporator section, the evaporation process is convinced to be uniquely contributing to the performance enhancement. It has been pointed out that the thermal performance enhancement of a nanofluid-charged TPCT is essentially attributed to the formation of nanoparticles deposition on the heated surface which modifies the surface wettability and enhances the nucleate boiling mechanism^35^,^36^,^37^,^38^. We analyze the strength of evaporation by evaluating the evaporator heat transfer coefficient augmentation ratio, *η*, as defined in Eq (2). Figure 5(b) plots *η* as a function of 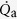 for different GO and SO concentrations. It can be observed that the average evaporator heat transfer coefficient, 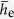, enhances in TPCTs with GO deposition, with a minimum of 11.4% and a maximum of 83.3% of enhancement as compared to the uncoated TPCT (charged with DI water). For SO deposition, no 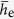 enhancement is observed for low SO concentration (0.01wt%) and even at a high SO concentration (0.5 wt%), the enhancement is relatively small compared to that of GO deposition. This discrepancy between TPCTs with GO and SO deposited layers is likely due to the distinct surface morphologies and characteristics of the depositions.

**Figure 6:**
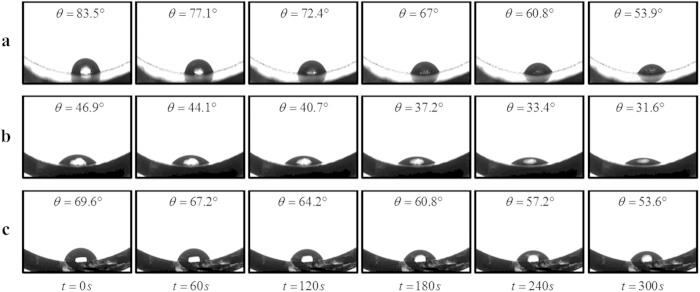
Time-lapse images of a 2 μl water droplet residing on (**a**) 0.1 wt% GO deposited layer, (**b**) 0.5 wt% SO deposited layer, and (**c**) uncoated glass surface, over a time span of 5 minutes. For each 60-s interval, the corresponding contact angle is recorded and depicted.

To this end, we investigate the surface wettability of deposited layer with a DI water droplet (2 μl) through measurement of its static contact angle on the substrate to observe how water spreads out. A low contact angle manifests high surface wettability which induces a higher evaporation rate as the liquid-solid contact area increases. Figure 6 displays the variations of contact angle on GO, SO deposited layers and uncoated glass surface over a time frame of 300 seconds. Although the GO deposition is the most hydrophobic (with the highest contact angle among the three cases), its decrease in contact angle over the time of 300 seconds is the highest with a rate of 0.099°/s. On the other hand, SO deposition which is the most hydrophilic (with the smallest contact angle) indicated a decrease in contact angle with a rate of 0.051°/s. For the uncoated glass surface, the contact angle decreases with a rate of 0.053°/s. The contact angle reduction rate of GO deposition is more than 1.9 times higher than that of SO deposition. As compared to the uncoated surface, the evaporation enhancement of SO deposited TPCTs is due the enhanced hydrophilicity of SO deposition. Of particular interest is the case of GO deposition. The anomalous enhancement in evaporation strength with GO deposited layer which is more hydrophobic than uncoated glass surface is contrary to the intuitive understanding. The factor of surface wettability is insufficient to explain this unusual phenomenon. In this case the unique fast water permeation property of GO comes into play. During the contact angle measurement of water droplet on GO deposition, for a longer time span, we observed that the contact angle continuously and gradually contracts over time and eventually the water droplet was completely absorbed into the GO deposited layer which is comparable to a sponge-like material. To ascertain the cause of the evaporation enhancement of GO deposition, in an individual experiment conducted under standard atmosphere, we compared the evaporation rates of water droplets on GO coated surface and uncoated surface with a surface temperature of 130^o^C (see Supplementary Movie M1). The evaporation rate of the former is 4 times of that of the latter, justifying the role of the fast water permeation in GO deposition in enhancing the evaporation strength of a heated surface.

**Figure 7:**
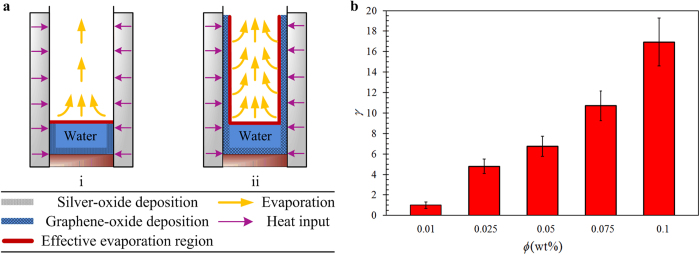
(**a**) Schematic illustration of evaporation process occurring at the effective region of a TPCT with (i) SO deposition, and (ii) GO deposition. In light of the fast water permeation effect, the effective evaporation region for TPCT with GO deposition is significantly extended across the evaporator wall surface (highlighted with red color) where filmwise evaporation is induced. (**b**) Relative deposition thicknesses of various GO nanofluid concentrations. The average thickness of 0.01 wt% GO deposition is used as a baseline for comparison.

Here we illustrate the nanoparticle depositions schematically. Referring to Fig. 7(a,i), The SO deposition is observed depositing in the submerged region and the effective evaporation region is only limited to the liquid-vapor interface. On the other hand, GO deposition spreads out across the evaporator wall above the liquid-vapor interface. The negatively charged hydroxyl groups at the edges of immersed GO sheets generate strong repulsive force between each individual GO sheet^45^. Concurrent with the upward liquid and vapor flows, the deposited GO sheets are spread across the wall surface covering substantially larger surface area. By virtue of water intercalation in the GO deposition, the effective evaporation region is extended to the wall surface where the GO sheets deposited, above the liquid-vapor interface (highlighted with red color) as depicted in Fig. 7(a,ii). As water intercalates between the GO interlayers, a thin film of water forms at the GO deposited layer. Evaporation occurs in a thin film is more effective than that in a pool of water due to larger surface area of the former. Even though it is in an antigravity direction, the water thin film formed at the GO deposition is continuously replenished from the pool of water through the operation of water permeation in the GO structure. In what follows, we denote the evaporation taking place at the GO deposited layer as filmwise evaporation.

**Figure 8:**
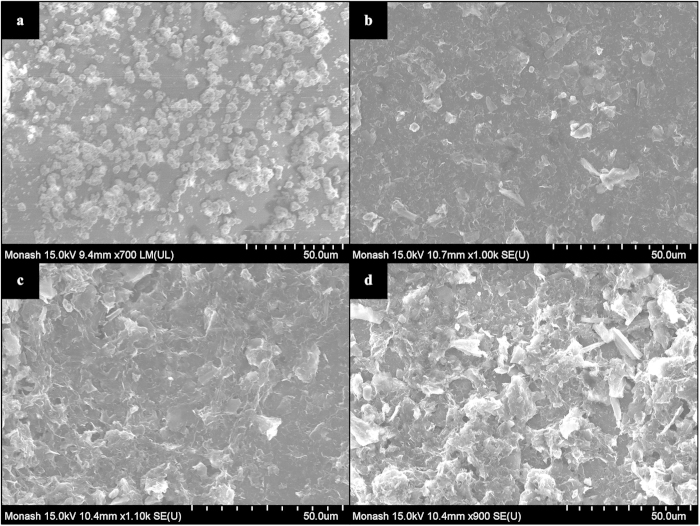
FESEM images of SO nanoparticles and GO sheets deposited on the glass surface of the evaporator section of TPCT: (**a**) 0.5 wt% SO nanoparticles, (**b**) 0.01 wt% GO sheets, (**c**) 0.05 wt% GO sheets and (**d**) 0.1 wt% GO sheets. The well distributed, closely-packed layered structure of GO sheets can be easily distinguished from the disorderly distributed spherical SO nanoparticles deposition. Higher concentration of GO sheets deposition is manifested in thicker strands.

To gain better insight into the mechanism of water permeation of GO deposition on the evaporator wall, we examine the surface morphologies of the deposited layers. FESEM was used to capture images of the deposited layers. Figures 8a,b,c,d illustrate the FESEM images of 0.5 wt% SO, 0.01 wt% GO, 0.05 wt% GO, and 0.1 wt% GO deposited layers, respectively. It is observed in Fig. 8(a) that spherically structured SO nanoparticles agglomerated and deposited disorderly on the surface. Although such deposition enhances the surface wettability, the enhancement of evaporation strength is not profound as discussed earlier. On the other hand, the GO depositions consist of well distributed, closely-packed layered structure of GO sheets which are responsible for the property of fast water permeation. For higher concentration of GO deposition (Fig. 8d), relatively thicker strands that can be easily distinguished from those of lower concentration (Fig. 8b) are observed. It is implied that the thickness of the GO layers depends on the nanofluid concentration.

To compare the GO deposition thicknesses of various nanofluid concentrations, we estimate the relative deposition thickness as *γ* = *t*_1_/*t*_0_, where *t*_1_ is the average thickness of GO deposition and *t*_0_ is the average thickness of 0.01 wt% GO deposition (lowest concentration) which is used as a baseline for comparison. The average thickness was approximated from the FESEM cross-section image of GO deposition which was then analyzed using the SigmaScan^®^ Pro (Systat Software, CA, USA) image processing software. For each concentration, a total of 15 cross-section images from 5 independent sets of experiment were captured. The average thickness of the baseline was estimated as 0.2 μm, corresponding to approximately 300 layers of GO sheets. Figure 7(b) depicts the relative deposition thickness of various GO nanofluid concentrations. The GO deposition thickness increases with nanofluid concentration and the thickness of 0.1 wt% GO deposition is 17 times of that of 0.01 wt% GO deposited layer.

It has been reported that water intercalation through GO membrane results in the expansion of the interlayer distance between the graphitic planes^16^,^18^,^19^. The X-ray diffraction (XRD) analysis of the hydrated GO membrane showed an increase in the interlayer distance from 7.7 Å to 12.29 Å^16^, capable of intercalating bilayer of water for fast water permeation^20^,^21^. Thus, the water permeability of GO membrane is appreciably enhanced when the interlayer spacing increases^16^,^21^. The variation of the interlayer spacing is temperature dependent^19^,^21^. Referring to Fig. 5(b), the 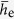 enhancement decreases with the increase in heat input and simultaneously the operating temperature that spans from 87 °C to 136 °C for the GO charged TPCT. The operating temperature is below the critical temperature of 200 °C where the interlayer spacing completely collapses for the minimum clearance of water monolayer (≈5 Å) due to excessive thermal annealing^19^. The decrease in the 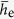 enhancement can be explained by the temperature variation which causes the structural transformation of GO deposition^19^, a phenomenon termed as thermohydration. As the temperature increases, structural alteration of the GO deposition takes place where the interlayer distance progressively decreases. The reduction in the interlayer distance greatly affects the water vapor permeability as less water monolayer is able to permeate through the narrowing gap. Thus, *η* reduction can be observed as the heat input increases.

In Fig. 5(b), it is obvious that two different regimes for the variation of *η* - Regime I 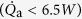 and Regime II 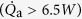 determine the evaporation enhancement for GO deposition. For a small 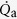 in Regime I, the 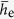 enhancement of TPCT with GO deposition decreases with both increasing 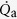 and GO concentration. This is intelligible that at low heat input, the evaporation rate at the liquid-vapor interface of the pool of water is relatively low while the filmwise evaporation from the extended GO thin film is vitally significant. Particularly for thinner deposited layer (lower GO concentration), the evaporation becomes more intense due to larger surface area. When the heat input increases, the contribution of evaporation at the GO deposited layer diminishes as its counterpart of the liquid-vapor interface intensifies. For thick deposited layer (high GO concentration), the effect of water permeation deteriorates, as elucidated in the following. In the oxidation process, GO sheets are attached by reactive oxygenated functional groups namely hydroxyl and carboxyl^41^ which enhance the hydrophilicity and act as a separation between two stacked GO sheets, allowing water molecules to travel between the interlayer of the two stacked GO sheets^12^. In regions with non-oxidized graphene sheets, fast water permeation prevails as water molecules slip through the atomically smooth carbon walls. When water molecules approach the oxidized regions with oxygenated functional groups, they are pinned down due to the strong hydrophilic nature of the functional groups, impeding the in-plane water permeation effect^12^,^14^,^46^. Stronger pinning effect prevails in thicker GO deposited layer. This explains the decreasing trend of *η* with increasing 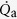 and the diminishing effect of GO deposited layer thickness on 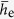 enhancement.

In Regime II for higher 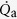, except for that of the thinnest GO deposited layer (0.01 wt%), *η* increases with 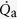 and approaches an asymptotic value at high heat input, as depicted in Fig. 5(b). At high heat input 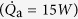, the 0.05 wt% and 0.025 wt% GO depositions (of middle thicknesses) manifest the highest *η*. This phenomenon is a compromise between the deteriorative effects on the evaporation strength associated with the thickness of GO deposition. When the deposited layer is too thin, the filmwise evaporation is too intense for water to be replenished through permeation in GO sheets to sustain the evaporation that dryout might take place and hinder the filmwise evaporation. As the evaporation at the liquid-vapor interface is relatively intense at high heat input, the significance of filmwise evaporation is not profound and therefore the evaporation enhancement due to the deposited layer is decreased. On the other hand, when the deposited layer is too thick, the fast water permeation deteriorates due to the water pinning effect of the hydrophilic functional groups, as discussed earlier. In this case, the effect of the thickness of GO deposition on the enhancement of evaporation is marginal at high heat input. Thus, the fast water permeation characteristics of the GO deposition which is intimately related to the structural alteration under temperature variation and deposition thickness are key factors in affecting the evaporation strength and hence the thermal performance of a GO/water nanofluid charged TPCT.

## Conclusions

In summary, we demonstrated the fast water permeation effect of immersed GO deposition on the evaporation strength of a GO/water nanofluid charged TPCT. The operation of fast water permeation in the nanocapillaries was attributed to the frictionless interaction between the atomically smooth, hydrophobic carbon wall and the well-ordered hydrogen bonds of the water molecules that gives rise to capillary force which is strong enough to overcome the gravitational force to form a thin water film on the GO deposited layers. The water intercalation induces the expansion of the interlayer distance between the graphitic planes which are temperature dependent. As a result, filmwise evaporation which is more effective than its interfacial counterpart is induced and the overall performance of TPCT is greatly enhanced. This study provides important insights into the mechanism of water permeation of immersed GO that exhibits an enormous potential in thermal management applications with special relevance to the development of two-phase cooling devices.

## Additional Information

**How to cite this article**: Tong, W.L. *et al.* Enhanced Evaporation Strength through Fast Water Permeation in Graphene-Oxide Deposition. *Sci. Rep.* **5**, 11896; doi: 10.1038/srep11896 (2015).

## Supplementary Material

Supplementary Information

Supplementary Movie M1
