# Supplementary material for: Enhanced Evaporation Strength through Fast Water Permeation in Graphene-Oxide Deposition
Source: Sci Rep. 2015 Jun 23;5:11896. doi: 10.1038/srep11896 (PMC4477327; doi:10.1038/srep11896)
Supplement: Supplementary Information [file srep11896-s1.pdf]

Supplementary Information for

Enhanced Evaporation Strength through Fast Water Permeation in Graphene-Oxide Deposition

Wei Li Tong[1], Wee-Jun Ong[2], Siang-Piao Chai[2], Ming K. Tan[1] and Yew Mun Hung[1]*

[1]Mechanical Engineering Discipline, [2]Multidisciplinary Platform of Advanced Engineering, Chemical Engineering Discipline, School of Engineering, Monash University, 46150 Bandar Sunway, Malaysia.

*Corresponding author. E-mail: hung.yew.mun@monash.edu

Supplementary Information:

Supplementary Figures S1 and Supplementary Movie M1 with legends are provided as follow.

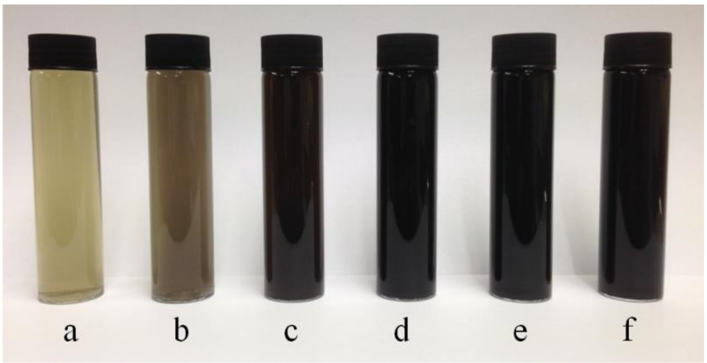

Figure S1. Digital images of nanofluids with various nanoparticles concentrations: (a) 0.01 wt% SO, (b) 0.01 wt% GO, (c) 0.025 wt% GO, (d) 0.05 wt% GO, (e) 0.075 wt% GO, and (f) 0.1 wt% GO.

Supplementary Notes for Movie M1

To quantitatively evaluate the permeability through GO deposition layers, in an individual experiment conducted under standard atmosphere, we compared the evaporation rates of water on a GO coated surface and an uncoated surface (see Supplementary Movie M1). To

prepare the GO coated surface, 30 ml of 0.1wt% GO nanofluid was applied to an aluminium surface heated at a temperature of 110°C. The solvent (water) is then evaporated leaving a uniform layer of GO deposition. To conduct the experiment, a 30 µl water droplet was dropped onto an uncoated surface (Case 1) and the GO coated surface (Case 2) which were both maintained at a surface temperature of 130°C (a typical wall temperature of the evaporator of TPCT). It was observed that due to permeability of water through the GO deposition, the water droplet evaporates much faster on the GO coated surface than on the uncoated surface. The water permeability of GO deposition significantly enhances the evaporation rate by rapidly spreading the water droplet. The respective time required for evaporation of a water droplet on the surface was calculated from the recorded videos. The estimated time for complete evaporation on the GO coated surface is 2.6 s while that on the uncoated surface reads 10.3 s. The evaporation rate, $\dot{m}$, is evaluated as $\dot{m} = m_{\mathrm{d}}/t$, where m_{d} denotes the mass of droplet and t is the estimated time. The evaporation rate of GO coated surface and uncoated surface are 13.9 mg/s and 3.5 mg/s, respectively. The evaporation rate of the former is 4 times of that of the latter, justifying the role of the fast water permeation in GO deposition in enhancing the evaporation strength.

Supplementary Movie Legend:

Supplementary Movie M1. Water droplet (30µl) evaporating on an uncoated surface (Case 1) and a GO coated surface (Case 2) with a surface temperature of 130°C. The droplet evaporation is significantly enhanced with the presence of the GO deposition. Fast water permeation of the GO deposition induces filmwise evaporation through the rapid spreading of the water droplet.
